# Shifts in Gait Signatures Mark the End of Lifespan in Mice, With Sex Differences in Timing

**DOI:** 10.3389/fnagi.2021.716993

**Published:** 2021-08-02

**Authors:** Lauren Broom, Jessica Stephen, Varun Nayar, Veronique G. VanderHorst

**Affiliations:** Division of Movement Disorders, Department of Neurology, Beth Israel Deaconess Medical Center and Harvard Medical School, Boston, MA, United States

**Keywords:** aging, walking, motor decline, balance, sex difference, gait signature, gait speed, longitudinal

## Abstract

Reduced walking speed is a hallmark of functional decline in aging across species. An age-related change in walking style may represent an additional key marker signifying deterioration of the nervous system. Due to the speed dependence of gait metrics combined with slowing of gait during aging, it has been challenging to determine whether changes in gait metrics represent a change in style. In this longitudinal study we employed gait signatures to separate changes in walking style and speed in mice. We compared gait signatures at mature adult age with middle aged, old and geriatric time points and included female and male sub-cohorts to examine sex differences in nature or timing signature shifts. To determine whether gait signature shifts occurred independently from a decline in other mobility domains we measured balance and locomotor activity. We found that walking speed declined early, whereas gait signatures shifted very late during the aging process. Shifts represented longer swing time and stride length than expected for speed, as in slow motion, and were preceded by a decline in other mobility domains. The pattern of shifts was similar between female and male cohorts, but with sex differences in timing. We conclude that changes in walking style, speed and other mobility domains represent separate age-related phenomena. These findings call for careful, sex specific selection of type and timing of outcome measures in mechanistic or interventional studies. The pattern of age-related gait signature shifts is distinct from patterns seen in neurodegenerative conditions and may be a translatable marker for the end of the lifespan.

## Introduction

Decreased gait speed is a major hallmark for dysfunction and physical frailty in old age (Fried et al., 2001; Ko et al., 2010; Buchman et al., 2014; Middleton et al., 2015; Herssens et al., 2018; Grande et al., 2019) and is a predictor for adverse outcomes such as disability, cognitive impairment, institutionalization, falls, and/or mortality (Abellan van Kan et al., 2009; Mielke et al., 2013; White et al., 2013; Veronese et al., 2018; Semba et al., 2020). Slowed gait has been associated with cardiovascular disease (Veronese et al., 2018), musculoskeletal problems (Bhasin et al., 2020), as well as disorders that specifically affect the central nervous system (CNS), including parkinsonism (Yogev et al., 2005), cognitive impairment and dementia (Visser, 1983; Tian et al., 2020), and small vessel disease (de Laat et al., 2010; Pinter et al., 2017). While gait speed is easy to determine, slowed gait as a global measure does not provide insights into the various factors that may contribute to this phenomenon.

**FIGURE 1:**
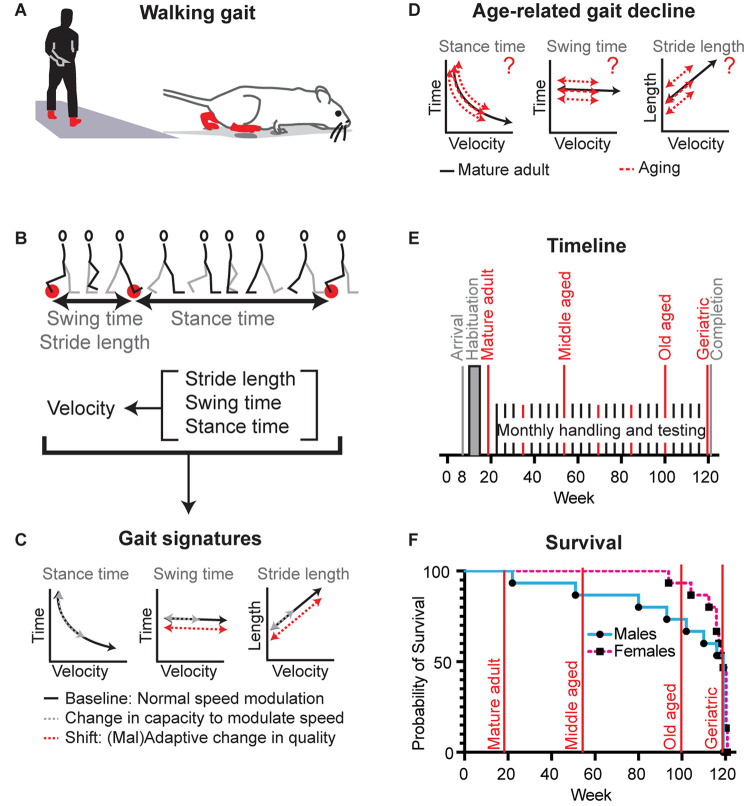
Definition of gait signatures and study design to distinguish age-related changes in walking speed from changes in walking phenomenology. **(A)** Walking represents a slow gait pattern that is shared between biped and quadruped models. **(B)** Determinants of walking velocity are the spatial metric stride length and the temporal metrics stance time and swing time. These three metrics change as a function of stride velocity, but do so in a distinct manner, each forming a distinctive signature among species (Broom et al., 2017, 2019). **(C)** During normal speed modulation (solid black lines) or a reduction in capacity to modulate speed (gray dashed lines), gait metrics abide the rules of these signatures. In the setting of adaptive or maladaptive changes in the nervous system, sets of gait signatures may shift [red dashed lines, example represents parkinsonism in human and mouse models (Broom et al., 2017, 2019)]. **(D)** It is not known whether age-related decline in gait speed involves changes capacity and/or walking style, what the timing and nature is of these changes, and whether they are the same for both sexes. **(E)** Timeline of the longitudinal study: Female (*N* = 15) and male (*N* = 15) cohorts of C57BL/6J mice entered the study simultaneously. We analyzed mobility and gait data at key time points (red lines) representing mature adult (19 weeks), middle aged (55 weeks), old aged (99 weeks), and oldest aged (119/120 weeks). **(F)** Survival curves of the female and male cohorts in relation to key time points.

A key question is whether age-related decline in gait speed is accompanied by changes in style (phenomenology) of walking. Traditionally, characteristic changes in walking style are recognized by trained clinicians via pattern recognition. This still forms the basis for clinical diagnosis (Giladi et al., 2013). However, this approach is subjective, hard to quantify and difficult to translate to (quadruped) models due to differences in size and anatomy. To separate changes in walking style from a decline in gait speed alone, measurements of spatial and temporal gait metrics can be helpful (Wilson et al., 2019). While there are many valid methods to capture gait metrics in human or animal models, the challenge lies in the interpretation of changes in gait metrics in the setting of declining speed: key gait metrics that dictate speed, i.e., stride length, stance time and swing time and their derivatives, change as a function of speed and do so independently (Figure 1; Broom et al., 2017; Mock et al., 2018). This problem is further magnified when datasets are averaged. For example, recent studies in mice reported age-related changes in average cadence (Bair et al., 2019; Tarantini et al., 2019) or the inverse, cycle duration (Mock et al., 2018). However, as gait speed declined from running and trotting speeds to trotting or slower speeds and as cadence decreases (or cycle duration increases) as a function of speed, it remains unclear whether these results were due to the decline in speed or represented a change in gait style. In more mechanistic animal studies, the concept of spatial-temporal gait metrics dictating walking style is underexplored. The majority of these studies have focused on control of gait speed and selection of (quadruped) gait patterns with emphasis on faster speed ranges that do not represent walking (Bretzner and Brownstone, 2013; Bouvier et al., 2015; Roseberry et al., 2016; Cabaj et al., 2017; Capelli et al., 2017; Correia et al., 2017; Caggiano et al., 2018). In aging adults, there has been an abundance of studies that report on gait or mobility, with a heavy focus on associations between gait variability, speed, falls, and executive functioning, e.g., (Springer et al., 2006; Callisaya et al., 2010; Wilson et al., 2019). Relatively few reports include gait metrics beyond velocity (Wilson et al., 2019) necessary for assessment of changes in style, but even when granular data are captured and sophisticated analyses are employed it has been challenging to detect distinct style patterns (Serrao et al., 2018). As a result, little is known about changes in walking style and underlying circuit mechanisms (Wilson et al., 2019).

That key gait metrics change as a function of gait velocity can be a barrier, but this feature can also be leveraged into a tool, i.e., by using this relation to define “gait signatures” that represent key elements of gait phenomenology (Akay et al., 2006; Broom et al., 2017, 2019; Figure 1). By establishing signatures at mature adult age, i.e., after development but before aging sets in, it then becomes possible to determine whether age-related changes in gait metrics stay true to or shift away from mature adult gait signatures. The former would implicate a change in capacity or drive and the latter a change in style. This approach has translational relevance, as the concept of gait signatures is conserved among quadruped and biped models (Hutchinson et al., 2006; Witte et al., 2006; Broom et al., 2017). We employed this methodology to show that walking style changes in patients with neurodegenerative disorders as well as in murine disease models (Neckel et al., 2013; Broom et al., 2017, 2019) and that distinct changes in walking style are the result of modulation of highly specific circuit nodes in the CNS [Worley, submitted].

Using this gait signature approach, the primary aim of this study was to determine whether age-related slowing of walking gait is accompanied by fundamental changes in gait style (Figure 1) in the commonly used C57BL/6J strain. We enhanced the translational relevance, by employing an adapted walkway to capture walking gait, rather than faster trotting and bound gaits which are commonly collected using generic rodent runways (Broom et al., 2017; Mock et al., 2018). To avoid the potential issue of sub-strains (Kelmenson, 2016), which would interfere with the interpretation of gait signatures (Broom et al., 2017), we followed the same mice up to the age of 28 months, the expected median survivorship of C57BL/6J mice (Hagan, 2017).

A secondary question to aide interpretation of the datasets involved the temporal relation between the decline in gait versus other mobility domains. Finally, as gait signatures differ between sexes at a very young age (Broom et al., 2017), we studied parallel cohorts of female and male mice to determine medium to large sex differences in nature or timing of age-related gait changes. The results demonstrated that age-related decline in gait speed, gait style, and other mobility domains occurred independently and in sex specific time windows.

## Materials and Methods

### Contact for Reagent and Resource Sharing

Further information and requests for resources should be directed to and will be fulfilled by the Lead Contact Veronique VanderHorst (vvanderh@bidmc.harvard.edu).

### Experimental Model

The Institutional Animal Care and Use Committee at Beth Israel Deaconess Medical Center reviewed and approved the experimental protocol (#007-2016). Handling and housing of animals, behavioral tests and euthanasia were performed according to the Guide for the Care and Use of Laboratory Animals at the animal research facility of the Center for Life Sciences at Beth Israel Deaconess Medical Center.

We used 15 female and 15 male C57Bl6/J mice (Jackson Laboratories; RRID: MSR_JAX:000664) which arrived simultaneously at the animal facility at 8 weeks old. Mice were group housed, except when separation was necessary due to fighting or toward the end of the study due to the loss of cage mates. Mice had access to standard enrichment, *ad libitum* water and food and were maintained in a temperature controlled environment on a 12 h light-dark cycle.

### Method Details

#### Behavioral Testing Conditions and Data Collection

Figure 1E outlines the timeline of the study. Male and female cohorts were purchased at the same time, housed in the same holding room, but tested on separate, consecutive days. We habituated and/or trained mice to each of the behavioral testing conditions, i.e., walkway, balance beam, open field arena for at least 4 sessions, starting at 10 weeks of age and continuing until week 12. Habituation was then maintained once per month throughout the study, with habituation in test months occurring the week prior to testing. Habituation occurred in the same rooms as testing. Behavioral tests were conducted every 2–4 months during the first half of the light-ON period and by the same handler (LB) as time of day (Silver et al., 1996) and handler (Broom et al., 2017) affect locomotor activity and gait speed, respectively. Testing was done 3 days or longer after bi-weekly cage changes given the impact of standard husbandry protocols on locomotor activity (Pernold et al., 2019). Throughout the study, all tests were performed in the same order (weight, gait, balance, and open field).

##### Weight

Weight was measured monthly using a digital scale (Fisher Scientific) at the time of habituation or on the day of the testing, and from age 99 weeks, we measured weights twice weekly as part of age appropriate routine health monitoring. Indications for euthanasia prior to the end of the study were weight loss of 20% and presence of tumors. The end point of the study was designed at 122 weeks (median 50% survival for male C57BL/6 mice). However, as weight and body condition declined rapidly after week 115, this time point was shifted to week 119 (males) and 120 (females) to maintain adequate group size for the primary test in this study, detailed gait analysis, at the geriatric time point.

##### Video gait assessment

Using high speed video, we obtained spatial and temporal gait parameters from hindlimb and forelimbs as described and validated in detail before (Broom et al., 2017, 2019). Briefly, foot print data was captured from video frames at 120 fps from a Casio Exilim FH-100 video camera, visualized on a 120 cm × 8 cm plexiglass walkway with a mirror placed underneath at an angle of 45 degrees, and aided by side view (Akay et al., 2006). As outlined before (Broom et al., 2017), the mouse-specific size of the runway prompts mice to walk rather than trot or run. This was crucial for the purpose of this study as generic “rodent” runways have larger dimensions which incite healthy young mice to trot or run rather than walk. These faster speeds are not relevant for translation and would also complicate comparison of datasets from (fast) young mice with (slow) older mice. At least 4 trials were recorded per mouse per session or more as needed to obtain a minimum of 4 valid trials, each containing at least 4 consecutive strides of continuous walking per limb.

##### Balance beam

We assessed balance with the horizontal beam test. We recorded mice with a conventional video camera (Sanyo Xacti WH1; 30 fps) while they walked across a 6 mm wide, 120 cm long beam (Carter et al., 2001). We obtained 2 trials per session in each mouse.

##### Open field

We assessed locomotor activity with the open field test. We recorded mice with a conventional video camera (30 fps) in a 80 cm × 80 cm × 50 cm box for 10 min.

#### Data Analyses

Behavioral tests were scored by raters blinded to the testing condition. Data from the mature adult life phase (week 19) served as baseline. Mice at this age have reached adult size (though not necessarily weight). Week 55 represented the middle aged life phase, and weeks 99 and 120 the old and geriatric aged life phases, respectively (Hagan, 2017). When appropriate, additional time points were analyzed. We used Graphpad Prism 8 software (San Diego, CA, United States) for statistical analysis. Family wise significance was set at 0.05, except for velocity dependent gait analysis, where it was set at 0.001 (Broom et al., 2017, 2019).

##### Survival curves

While the study was designed to end at 122 weeks, the estimated 50% survivorship for C57BL/6J males (Jackson Laboratories), it had to be ended earlier as weight and condition started to drop in individual mice after week 115, prompting humane euthanasia. Mice were euthanized for the presence of tumors or weight loss at pre-set standard criteria or if no health issues occurred at the end of the study (week 119 for males, week 120 for females). Both terminal time points are represented as “week 120” for simplification. In both male and female groups, we reconstructed survival curves.

##### Weight

Data sets were tested for normality using the Shapiro–Wilk test and we then used Repeated Measure-one-way-ANOVA to compare weight at baseline (19 week) and other time points, followed by Dunnett’s multiple comparison tests as appropriate.

##### Video gait analysis

We used a validated approach to analyze gait metrics that were captured on video (Akay et al., 2006; Broom et al., 2017, 2019). Spatial (location of the footfalls) and temporal data (time stamps of stance and swing phases) were extracted from video frames using custom MATLAB code to obtain the following parameters: stride velocity, swing and stance time, stride length, cadence, and swing speed (Broom et al., 2017). Briefly, for each stride we measured swing and stance durations derived from video frame number and determined stride length from spatial coordinates using MATLAB. We derived stride velocity, cycle duration (sum of stance and swing durations), and cadence (1/cycle duration) from these measures. Gait metrics were plotted against their stride velocity, errors were removed and we then applied regression models, capturing the speed range that represents walking, i.e., 3–16 cm/s (Broom et al., 2017). These data represent gait signatures. Using a extra sum-of-squares *F* test we then determined whether regression curves (i.e., gait signatures) that summarize datasets at different time points within the male and female cohorts were shared, or whether male and female datasets at the same time points were shared. This approach has the advantage that it takes into account the entire model, not only intercept or slope. While gait signatures can be reconstructed for both fore- and hindlimbs (Broom et al., 2019), here we focus on hindlimb metrics as these are most important for propulsion and are most relevant for human gait comparisons. To provide insight in animal to animal variability, we supplemented this with a comparison of averaged data sets at baseline (19 weeks) and at aged time points using RM-one-way-ANOVA, followed by Dunnett’s multiple comparison tests as appropriate. Data passed normality testing using the Shapiro–Wilk test and linear data sets were screened for outliers (ROUT method with *Q* = 1% leading to 0–13 outliers/2,087 data points per metric).

##### Balance beam

We quantified numbers of slips and misses of the hind paws from the video recordings and calculated average performance of 2 trials per session in each mouse. Data sets were tested for normality using the Shapiro–Wilk test and we then used RM-one-way-ANOVA to compare baseline (19 week) performance and other time points, followed by Dunnett’s multiple comparison tests as appropriate. Group size at aged time points was dictated by the number of mice able to perform this test.

##### Open field

We quantified time spent immobile and overall distance traveled from the video recordings. Data sets were tested for normality using the Shapiro–Wilk test and we then used RM-one-way-ANOVA to compare baseline (19 week) performance and other time points, followed by Dunnett’s multiple comparison tests as appropriate.

**FIGURE 2:**
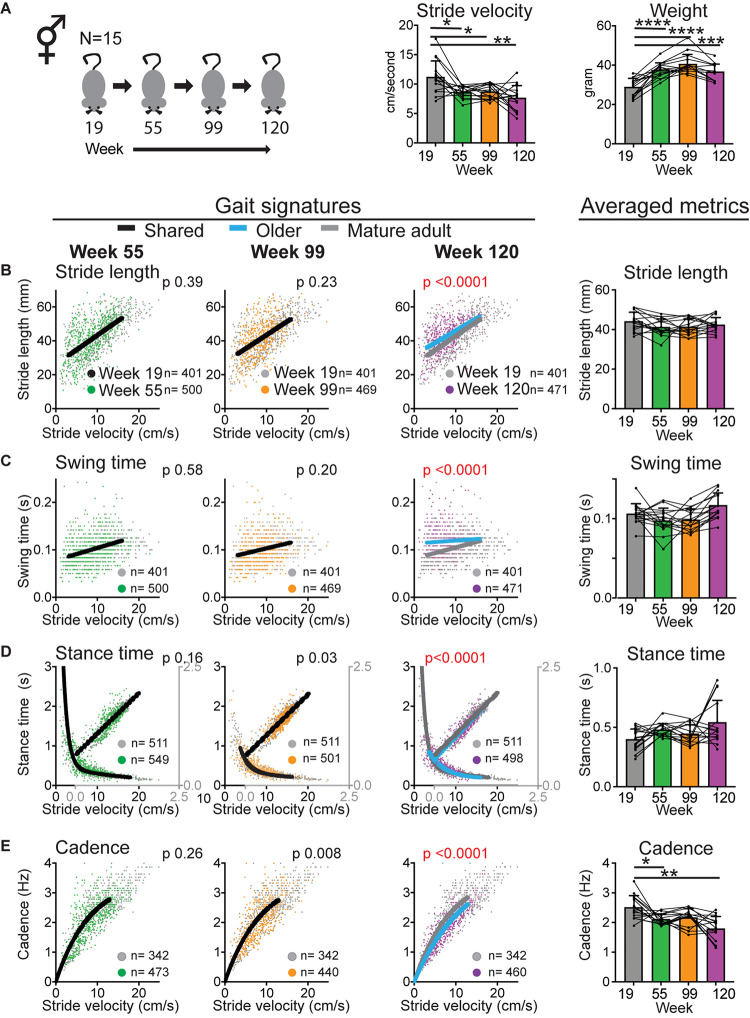
Walking velocity declines early whereas gait signatures shift at geriatric age. **(A)** Bar graphs of average stride velocity from uninterrupted walking bouts on a walkway and weight at key time point during aging (mixed cohort *N* = 15). Error bars indicate standard deviation. RM-one-way ANOVA followed by Dunnett’s multiple comparison test with correction for multiple comparisons; family alpha 0.05. **(B–E)** Stride by stride gait metrics stride length **(B)**, swing time **(C)**, stance time **(D),** and cadence **(E)** plotted as a function of stride by stride velocity. Gray dots represent data from week 19 (mature adult) whereas green, orange, and purple represent data from weeks 55, 99, and 120, respectively. Single black curves or lines indicate that the dataset from week 19 and key time points fit the same curve, whereas separate lines indicate that datasets are significantly different (Sum of square *F*-test; alpha 0.001). Note the shift in curves or gait signatures (blue lines) in the geriatric age group (week 120). Bar graphs to the right show averaged metrics to illustrate variation between animals and time points. Error bars indicate standard deviation. RM-one-way ANOVA followed by Dunnett’s multiple comparison test with correction for multiple comparisons; family alpha 0.05. Curves were fitted for the velocity range that represents walking gait. *N*, number of mice; *n*, number of strides included in the analyses. Statistical analyses are summarized in Supplementary Table 1. *, **, ***, and **** represent *p* values of < 0.05, < 0.01, < 0.001, and < 0.0001, respectively.

**FIGURE 3:**
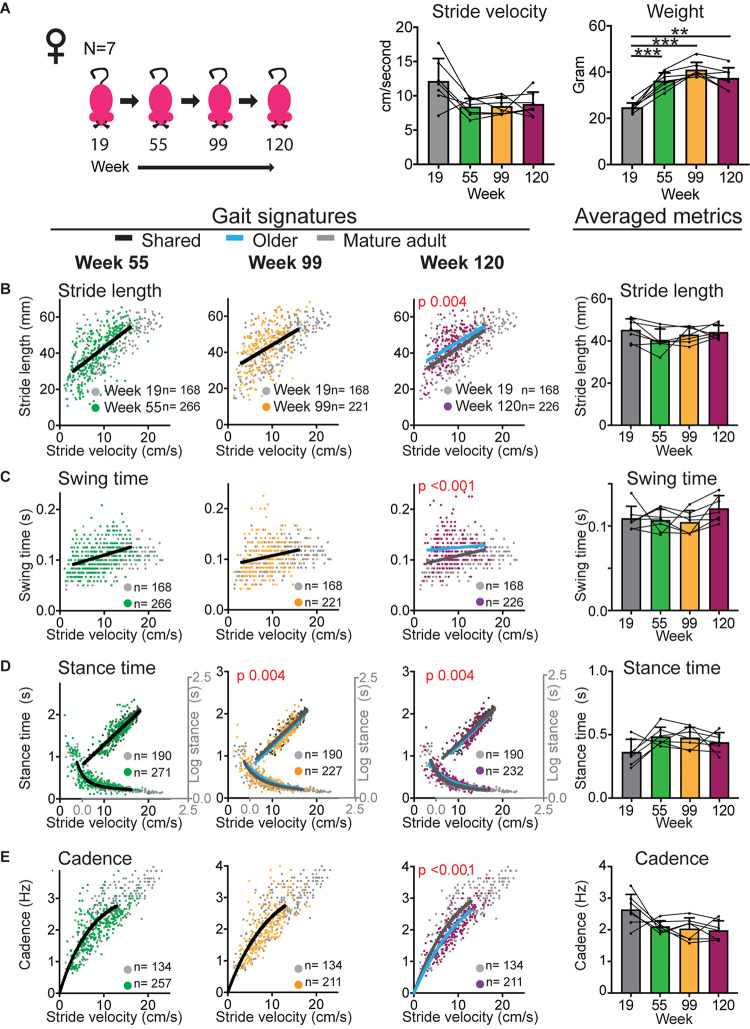
Gait signatures in female mice shift at the geriatric time point without an accompanying decline in walking speed. **(A)** Bar graphs of weight and average stride velocity from continuous walking bouts on a walkway at key time points in the sub-cohort of female mice that reached the 120 week end point. Error bars indicate standard deviation. RM-one-way ANOVA followed by Dunnett’s multiple comparison test with correction for multiple comparisons; family alpha 0.05. **(B–E)** Stride by stride gait metrics stride length **(B)**, swing time **(C)**, stance time **(D)**, and cadence **(E)** plotted as a function of stride by stride velocity. Gray dots represent data from week 19 (mature adult) whereas green, orange and purple represent data from weeks 55, 99, and 120, respectively. Single curves or lines indicate that the dataset from week 19 and older time points fit the same curve, whereas separate lines indicate that datasets cannot be fitted in the same model and are significantly different (Sum of square *F*-test; alpha 0.001). Note the shift in curves or gait signatures (blue lines) in the geriatric age group (week 120). Bar graphs to the right show averaged metrics to illustrate variation between animals and time points. Error bars indicate standard deviation. Curves were fitted for the velocity range that represents walking gait. *N*, number of mice; *n*, number of strides included in the analyses. RM-one-way ANOVA followed by Dunnett’s multiple comparison test with correction for multiple comparisons; family alpha 0.05. Statistical analyses are summarized in Supplementary Table 2. ** and *** represent *p* values of < 0.01 and < 0.001, respectively.

**FIGURE 4:**
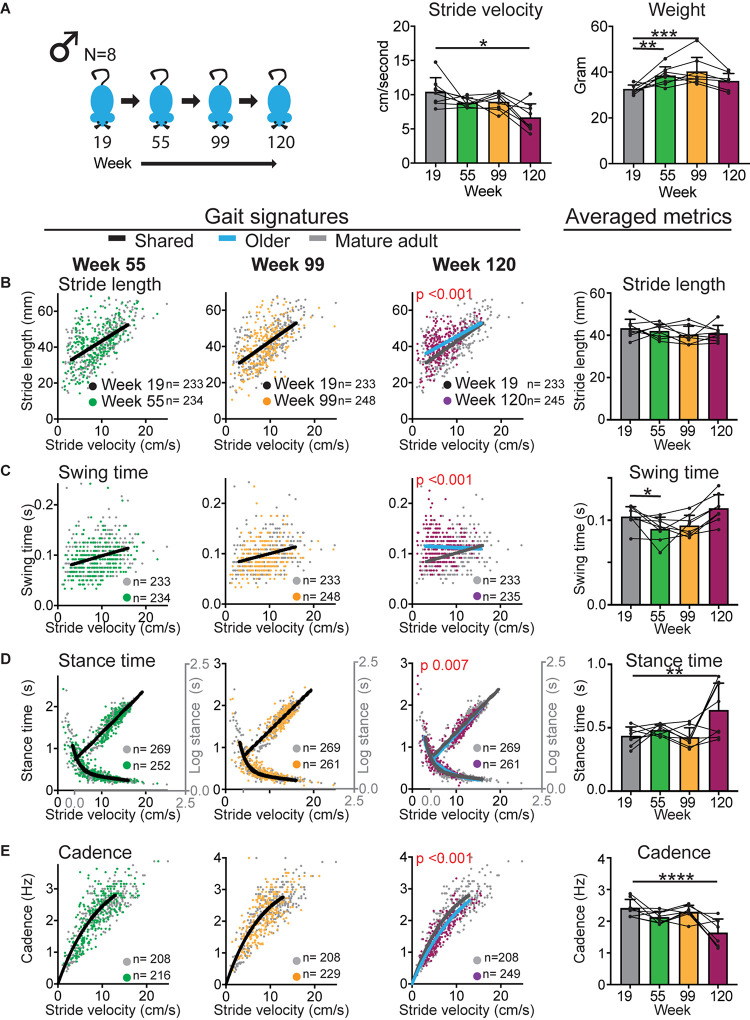
Gait signatures in male mice shift at the geriatric time point with an accompanying decline in walking speed. **(A)** Bar graphs of weight and average stride velocity from continuous walking bouts on a walkway at key time points in the sub-cohort of male mice that reached the 120 week end point. Error bars indicate standard deviation. RM-one-way ANOVA followed by Dunnett’s multiple comparison test with correction for multiple comparisons; family alpha 0.05. **(B–E)** Stride by stride gait metrics stride length **(B)**, swing time **(C)**, stance time **(D)** and cadence **(E)** plotted as a function of stride by stride velocity. Gray dots represent data from week 19 (mature adult) whereas green, orange and purple represent data from weeks 55, 99, and 120, respectively. Single curves or lines indicate that the dataset from week 19 and older time points fit the same curve, whereas separate lines indicate that datasets cannot be fitted in the same model and are significantly different (Sum of square *F*-test; alpha 0.001). Note the shift in curves or gait signatures (blue lines) in the geriatric age group (week 120). Bar graphs to the right show averaged metrics to illustrate variation between animals and time points. Error bars indicate standard deviation. Curves were fitted for the velocity range that represents walking gait. *N* = number of mice; *n* = number of strides included in the analyses. RM-one-way ANOVA followed by Dunnett’s multiple comparison test with correction for multiple comparisons; family alpha 0.05. *, **, ***, and **** represent *p* values of < 0.05, < 0.01, < 0.001, and < 0.0001, respectively. Statistical analyses are summarized in Supplementary Table 3.

**FIGURE 5:**
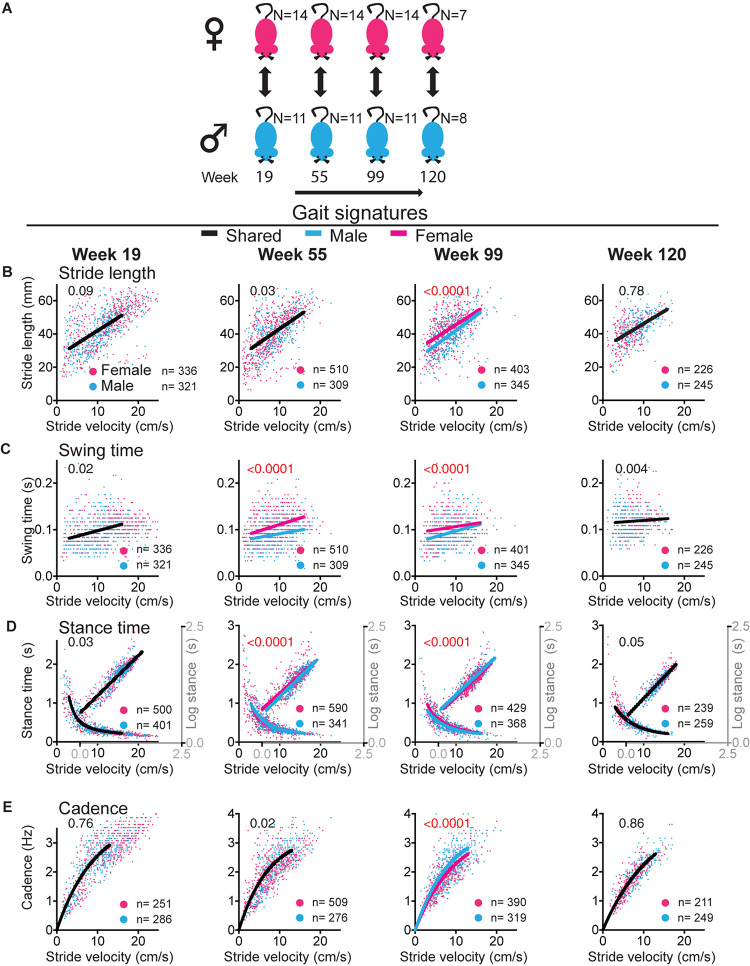
Sex differences in gait signatures during aging. **(A)** Experimental paradigm for comparisons between female (magenta) and male (blue) mice. **(B–E)** Stride by stride gait metrics stride length **(B)**, swing time **(C)**, stance time **(D)**, and cadence **(E)** plotted as a function of stride by stride velocity. Single curves or lines indicate that the female and male datasets fit the same curve, whereas separate lines indicate that datasets are significantly different (Sum of square *F*-test; alpha 0.001). Note that males and females share curves at the mature adult (week 19) and geriatric (week 120) time points, but that sex differences exist in swing time at middle age (week 55) and all metrics at old age (week 99). Curves were fitted for the velocity range that represents walking gait. *N*, number of mice; *n*, number of strides included in the analysis. Statistical analyses are summarized in Supplementary Table 4.

**FIGURE 6:**
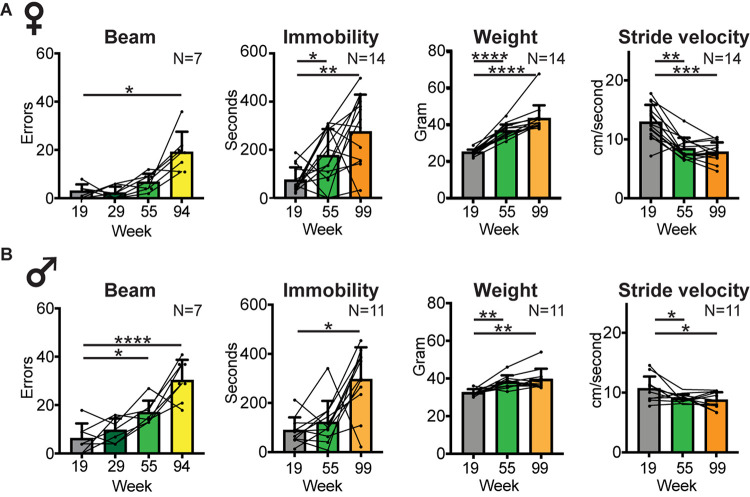
Balance and locomotor activity decline in the middle aged to old age time range. Bar graphs of average number of slips and misses while traversing a horizontal balance beam, and average time spent immobile in an open field arena (as a measure of locomotor activity) at key time points in female **(A)** and male **(B)** cohorts. Group size and time points were dictated by the inability of mice to perform the test at later time points. Average body weight and average stride velocity of the same mice are included for full transparency. Error bars indicate standard deviation. RM-one-way ANOVA followed by Dunnett’s multiple comparison test with correction for multiple comparisons; family alpha 0.05. Statistical analyses are summarized in Supplementary Table 5. *, **, ***, and **** represent *p* values of < 0.05, < 0.01, < 0.001, and < 0.0001, respectively.

In secondary analyses of various mobility measures and gait speed, we used 2 way ANOVA to assess for sex differences in performance during aging. For correlational analyses between gait measures and mobility measures, we first determined *Z* scores for each measure based upon the performance of the cohort at mature adult age. We then correlated *Z* scores at the time point of poorest performance (gait measures at 120 weeks, beam at 55 weeks, and locomotor activity at 99 weeks) using Pearson correlation. Details of primary and supportive analyses are summarized in Figures 2–6 and accompanying Supplementary Tables 1–7.

#### Power Calculations

The design of this study was optimized to reduce variation by using the same mice throughout the study, by starting at mature age, i.e., following completion of skeletal growth which can be a covariate (Akula et al., 2020), and by testing of parallel male and female cohorts under the same experimental conditions and by the same staff. The primary goal of this study was to detect medium (Cohen *d* = 0.5) to large (Cohen *d* = 0.8) age-related effects, with as secondary goal to assess for medium to large size sex differences. For standard metrics, using ANOVA, with an alpha of 0.05 and beta of 0.95, with 2 groups (male and female) and 4 measurements (19, 55, 99, and 120 weeks), to detect a large effect size a sample size of 6 yields a power of 0.85–0.95; to detect a medium effect size a sample size of 8 yields 85% power. We estimated to lose mice at the tail end of the study based upon available survivorship data, and therefore we started out with larger cohorts of 15 mice per sex. For the extra sum of square F test used for gait analyses, *post hoc* analyses revealed that with an alpha of 0.001, numerator df 2, denominator df ranging from 456 to 927, and λ ranging from 23 to 24, the power ranged from 0.87 to 0.90% [G^∗^Power; (Faul et al., 2007, 2009)].

## Results

### Survivorship

We followed 15 female and 15 male C57BL/6J mice from mature adult age up to the age of 120 weeks as outlined in Figure 1. Survivorship dictated the number of mice remaining at each time point and loss of mice was taken into account in the design of the study. Median survival was 115 weeks for the female and 93 weeks for the male cohort (Log rank Mantel-Cox test; Chi Square 4.014; p 0.045). At key time points, i.e., mature adult (week 19), middle aged (week 55), old aged (week 99), and geriatric (120 weeks), 15, 15, 14, and 7 females survived, respectively. In the male group, 15, 13, 11, and 8 mice survived at the respective time points. Several mice reached criteria for humane euthanasia prior to week 120 due to tumor growth (*n* = 4 males at ages 81, 94, 103, 111 weeks; *n* = 1 female at 117 weeks) or weight loss [*n* = 1 male at age 118 weeks, *n* = 6 females at ages 94, 104, 116 (*n* = 2), 119 (*n* = 2) weeks]. One female (age 112 weeks) and two male (ages 22 and 51 weeks) mice were found dead unexpectedly. Power analyses (see section “Materials and Methods”) show adequate power to detect medium to large size differences between time points or sexes. We will first present data from the mixed cohort that reached the 120 week end point to answer the primary question of whether gait signatures change in mice during aging. We will then analyze the nature and timing of changes within and between the male and female cohorts.

### Age-Related Changes in Gait Speed and Shifts in Gait Signatures

To confirm that walking speed decreases in mice during aging and to determine the temporal course of this decline, we measured gait speed at key time points (Figure 1). We derived speed from stride to stride metrics during continuous walking bouts on a walkway. The walkway was designed to capture walking gait rather than faster trotting or running gaits as described before (Broom et al., 2017). This facilitates comparisons within the same gait domain (i.e., walking) between young and old mice, rather than faster gait in young mice and walking gait in older mice. We found that speed decreased significantly [Figure 2; RM-ANOVA; *N* = 15; *F* (2.047, 38.21) = 9.047; and *p* < 0.0006]. *Post hoc* analyses showed that changes occurred as early as at the middle aged time point and remained significant throughout the aging process (Supplementary Table 1).

The main question is now whether gait metrics abide by the rules of gait signatures that are established at the mature adult stage or whether gait signatures shift during aging. To assess this we used a validated approach to construct walking signatures of gait metrics that dictate gait speed, i.e., the spatial metric stride length (distance between one footfall and the next of the same limb), the temporal metrics stance time (time foot makes contact with the ground), and swing time (time foot is off the ground) and their inverse combined metric, cadence (i.e., turnover of the feet expressed in Hz; Broom et al., 2017, 2019). We first determined baseline gait signatures at the mature adult stage (19 weeks) and, by following the same mice longitudinally, determined whether gait signatures at key aging time points shared the 19 week baseline signature using an extra sum of square *F* test. Gait signatures for stride length, swing time, stance time and cadence remained stable at the middle aged and old time points, but shifted significantly at the geriatric time point (Figure 2 and Supplementary Table 1). Stride length, swing time and stance time were longer than expected for speed, whereas cadence was reduced. Note that the averaged gait metrics, which do not separate changes in gait metrics that are due to the decrease in speed itself from changes in basic rules (signatures), do not yield significant changes except for cadence (Figure 2 and Supplementary Table 1). A likely explanation for this is that age-related changes in gait metrics are opposite changes that occur in the setting of decreasing speed alone, except for cadence. These findings of changes in gait speed occurring early and shifts in gait signatures occurring indicate that these phenomena are dissociated during aging.

### Are the Patterns of Age-Related Gait Changes Similar for Male and Female Mice?

In both female and male cohorts stride velocity decreased between baseline and middle age or old age time points [RM-one-way ANOVA: *N* = 14 females *F* (1.389, 18.06) = 31.51; *p* < 0.0001; *N* = 11 males *F* (1.655, 16.55) = 6.203; *p* 0.013; see also Figure 6]. This decrease was more striking in females who had a larger baseline speed than males. At the 120 week time point, speed declined in the male group [RM-one-way ANOVA: *F* (3, 21) = 7.326; 0.002; Dunnett’s *post hoc* 19 versus 120 weeks *p* 0.0004] but not in the female subcohort that reached that age [RM-one-way ANOVA: *F* (1.505, 9.031) = 4.68; *p* 0.05; Dunnett’s *post hoc*: 19 versus 120 weeks *p* 0.23; Figures 3, 4; Supplementary Tables 2, 3].

Based upon the different temporal course of age-related speed decline in male and female mice, the question is now whether shifts in gait signatures occur earlier in females than males, in parallel with the timing of speed changes, or whether the decline in walking speed and shifts in gait signatures are dissociated as suggested by analyses of the mixed cohort. In females (Figure 3 and Supplementary Table 2), despite the sharp reduction in average speed from 12 to 8 cm/s between mature and middle aged time points, both the cohort that reached week 99 (*N* = 14) as well as the subcohort that reached the 120 week time point (*N* = 7) did not reveal shifts of gait signatures at the middle aged time point (week 55). At the 99 week time point, we detected a small but statistically significant change in stance time in both the large and small sub-cohorts. At the 120 week time point, significant changes were present in all gait metrics in the same direction as for the mixed cohort.

In the male cohort (Figure 4 and Supplementary Table 3), analyses of the cohort that reached week 99 (*N* = 11) and the sub-cohort that reached the 120 time point (*N* = 8) revealed no gait signature shifts at the middle aged (week 55) and old aged (week 99) time points. At the 120 week time point, significant changes were present in all gait metrics in the same direction as for the mixed and female cohorts. These analyses show that the nature of gait signature shifts are similar in female and male cohorts. These results further support the hypothesis that shifts in gait signatures, which signify a change in walking style, can be dissociated from changes in walking speed.

### Gait Signatures at Mature Adult and Geriatric Times Points Are Shared Between Female and Male Mice but Differ at Other Time Points

While patterns of changes in gait signatures are similar between males and females, the question remains of whether there are sex related differences in gait signatures at key time points. This question is relevant, as we showed that gait signatures in younger adult female and male mice as well as older human subjects are dissociated, with a shorter stride length and increased cadence in females (Broom et al., 2017). We found that at the mature adult age (age 19 weeks), female and male mice shared gait signatures (Figure 5 and Supplementary Table 4). At week 55, gait signatures for swing time were dissociated with a larger swing time in females than males. At 99 weeks all signatures were dissociated, with larger stride length, swing time and stance time and decreased cadence in females. At the end point of 120 weeks, female and male cohorts had shared gait signatures. These data suggest that the development of shifts in gait signatures may differ between sexes, occurring earlier in females, but with the process of shifting signatures completed in both sexes around week 120. Interestingly, the direction of sex related shifts during the aging process are opposite in direction to the difference between males and females at the young adult age.

### Does the Decline in Other Age-Related Mobility Measures Follow the Time Course of Declining Gait Speed or Shifts in Gait Signatures?

To determine whether other mobility measures followed the same temporal pattern of decline as gait speed or shifts in gait signatures, we measured balance (ability to move across a horizontal beam without slips or misplacements of the feet) and spontaneous locomotor activity (immobility in an open field; Figure 6, Supplementary Table 5). Performance on the beam declined at middle age in both female and male cohorts, and by 94 weeks (1 month prior to key time point 99) only 7 female and 7 male mice were able to perform this test and did so with a significantly higher number of errors [RM-one-way ANOVA: females *F* (1.603,9.615) = 16.79, *p* < 0.001; males *F* (1.603,9.615) = 16.79, *p* < 0.001]. This number declined further, prohibiting meaningful comparisons at later time points. At the 120 week time point none of the mice were able to perform this test. Secondary analyses showed a sex difference in beam performance only at the middle aged time point, with males making more mistakes [2-way-ANOVA, *F* (1,23) = 5.51, *p* 0.03; Supplementary Table 6].

Locomotor activity also started to decline at middle aged or old age, with increased time spent immobile [RM-one-way ANOVA: *N* = 14 females *F* (1.837, 23.89) = 9.456; *p* 0.001; *N* = 11 males *F* (1.282, 12.82) = 12.1; *p* 0.002]. Secondary analyses showed no sex difference in time spent immobile in the open field at any time point [2 way ANOVA, *F* (1,23) = 0.076; *p* 0.78; Supplementary Table 6].

As weight may affect motor performance, we measured weights throughout the study. Weight increased from mature adult to middle aged time points and was then maintained up to old age [week 99; RM-one-way ANOVA: *N* = 14 females *F* (1.263, 16.42) = 70.8; *p* < 0.0001; *N* = 11 males *F* (1.515, 15.15) = 14.31; *p* 0.0007]. At the 120 week time point, weight alone was unlikely to drive the changes in gait curves (Figures 3, 4) as 120 week weight remained similar to middle aged weight in females, and was similar to baseline in males [week 120; RM-one-way ANOVA: *N* = 7 females *F* (2.025, 12.15) = 47.62; <0.0001; *N* = 8 males *F* (3, 21) = 8.698; *p* 0.0006]. Secondary analyses showed a sex difference in weight only at the (youngest) mature adult time point, with females weighing less [2 way ANOVA, *F* (1,23) = 1.75, *p* < 0.0001; Supplementary Table 6].

Finally, we assessed whether age-related changes in gait speed, swing time, balance and locomotor activity, expressed as *Z* scores that represent the change between baseline and time point of poorest performance (walking speed and swing time: 120 weeks; beam: 55 weeks; and locomotor activity: 99 weeks) correlated with each other. Changes in these performance measures did not correlate with each other (Pearson correlation; *N* = 15; Supplementary Table 7).

## Discussion

**FIGURE 7:**
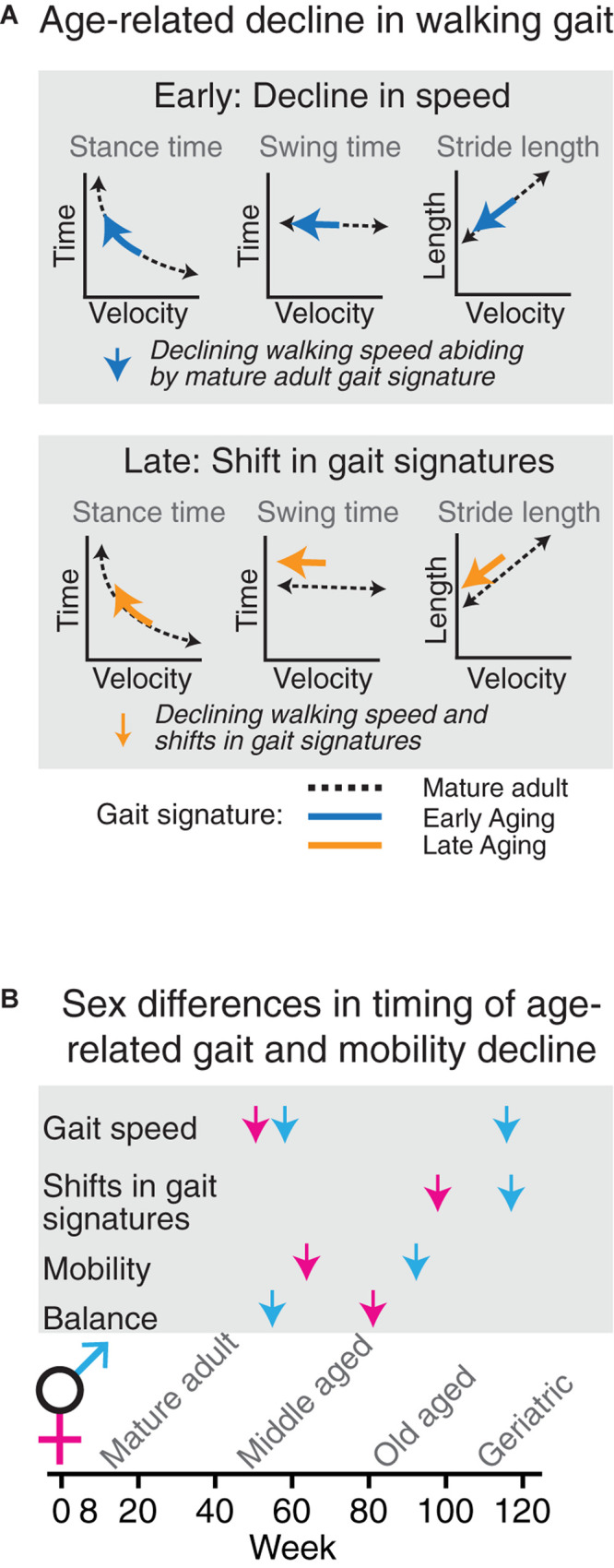
Age-related decline in walking gait occurs in two phases which are temporally dissociated from age-related decline in other measures of mobility. **(A)** Speed of overground walking decreases early (blue arrows), whereas shifts in gait signatures (orange arrows) signifying a change in gait phenomenology occur at the end stages of the aging process. **(B)** In aging female (magenta) and male (blue) C57BL/6J mice, changes in gait speed, gait signatures, locomotor activity and balance are temporally dissociated.

We set out to answer the question of whether age-related decline in walking gait speed in mice is accompanied by changes in walking style. We employed gait signatures to separate speed and style dimensions of walking gait in aging mice, which to the best of our knowledge has not been reported before. We found that despite an early decline in walking speed and other mobility domains, gait signatures were stable well into old age (Figure 7). Gait signatures then shifted at the geriatric time point, with swing time and stride length being longer than expected for speed. These shifts, as well as a decline in other mobility domains, were of the same nature in female and male cohorts, but with sex differences in timing. The temporal dissociation of the decline in different mobility domains points to separate mechanisms, with additional sex specific difference in vulnerability for each domain. Timing is therefore a critical variable in studies aiming to unravel mechanisms underlying each of these age-related changes.

The finding of signature shifts in aging mice has several implications. First, the results suggest that mechanisms driving the decline in age-related gait speed early in the aging process are different from mechanisms that drive shifts in gait signatures late in the process. Indeed, components that contribute to signature shifts involve (mal)adaptive changes in highly specific brainstem circuits that control the respective gait metrics [Worley, submitted] or in upstream circuits (Broom et al., 2017). In contrast, components contributing to a reduction in gait speed alone may be due to a decline in cardiorespiratory (Veronese et al., 2018) and musculoskeletal function (Bhasin et al., 2020) and in neural circuits that control cognition, motivation or anxiety (Visser, 1983; Lok et al., 2013; Tian et al., 2020), or gain of motor systems such as the medullary serotonergic system [Worley submitted].

Secondly, the pattern of age-related shifts in gait signatures in this study is distinct from the various patterns seen in different models of experimental parkinsonism (Broom et al., 2017) or in ataxia (Machado et al., 2015). For example, signatures of stride length and swing time shift upward in aging and downward in MPTP induced experimental parkinsonism. Cadence (a measure derived from both stance and swing time) decreases during aging but increases in MPTP induced experimental parkinsonism, and stance time increases during aging but decreases in ataxic mouse lines. As weight in the MPTP cohort did not differ between baseline and experimental conditions at the time of testing (Broom et al., 2017) differences within or between models are unlikely the result of changes in weight alone. Instead we speculate that these differences in shifts in gait signatures between age-related and neurodegenerative experimental models are due to differences in circuit dysfunction between these models. In other words, age-related shifts in gait signatures are likely due to changes in neural circuit function, but affected circuits differ from those manipulated in the few experimental neurodegenerative models that have been tested so far (Broom et al., 2017). Indeed, using chemogenetic approaches to temporary (hours) modulate discrete, cell type specific circuit nodes in the caudal brainstem, we have been able to elicit different gait signature shifts [Worley submitted]. Further studies will be needed to identify these circuits and the nature of the dysfunction.

Next, this study demonstrated that age-related decline in gait speed and shifts in gait signatures occur temporally separate from each other and from age-related decline in locomotor activity and balance. Furthermore, changes in performance in these metrics did not correlate with each other. As such, each of these metrics represent distinct modalities of age-related changes in mobility and are not suitable as proxy for each other.

Finally, this study provides insights in sex differences in the nature and timing of age-related changes in gait and mobility. We reduced the impact of variability by testing male and female cohorts in a parallel longitudinal design under the exact same experimental conditions. Prior studies focused on male mice (Bair et al., 2019; Tarantini et al., 2019) or combined cohorts (Zhang et al., 2014). One study in which male and female mice in different age groups were compared did not reveal sex differences in average speed, cycle duration (sum of swing time and stance time), base or swing speed, but that study did not have a longitudinal design and mice were being followed up to old age only (17 months; Mock et al., 2018). Furthermore, the latter study did not control for changes in speed, as discussed above. We found that while the nature of changes in walking gait was similar in male and female aging cohorts, the timing of decline differed for gait speed (prominent early in females due to a higher baseline speed and prominent in geriatric males but not females), for gait signature shifts (deviating between females and males at middle aged and old age suggesting smaller shift in males but converging at geriatric ages) and for balance (earlier in males).

The findings in this study lead to questions as to what underlies these sex related differences in timing for each of the mobility domains, which can now be examined in follow up studies using the appropriate time windows. They also raise a more practical question: should age-related changes be studied in mixed or sex specific groups? This depends on the question and metric being examined. The direction and endpoint of age-related changes in all measures included in this study was similar in female and male cohorts. Therefore, if the central question is related to the presence or size of the changes at the aging end points, the use of mixed groups is warranted. However, as decline of the various mobility measures develops during specific time windows, using separate female and male cohorts will be important if the focus is on the development of underlying pathophysiology.

### The Impact of Study Design on Interpretation of Gait Datasets That Capture Age-Related Changes

Separating modulation of walking speed from modulation of walking style through analyses of gait metrics provides a coarse but important step in elucidating underlying mechanisms as outlined above. The methodology employed in this study enabled analysis of gait metrics in a speed dependent manner, facilitating separation of speed-related changes from speed-independent modulatory changes even in the setting of decreasing speed. In contrast, prior aging studies analyzed gait metrics as averaged datasets, which provided insights in changing metrics but without the granularity necessary to determine whether these changes were normal for the change in speed or reflected pathological gait.

More specifically, for some metrics, averaged data pointed in the same direction as in the present study. This was the case for cadence (Bair et al., 2019; Tarantini et al., 2019). However, as is obvious from the gait signature of cadence (Figure 1), cadence decreases rapidly when gait speed declines. Thus, if speed changes sufficiently average cadence will drop as a consequence. We illustrated this in complementary averaged datasets in which we detected a decrease in average cadence in the male cohort in which speed declined significantly at the latest time point, but not in the female cohort in which speed remained stable from middle aged to oldest aged time point.

The use of averaged data may also be one of the reasons that changes in step length were not detected by Bair et al. (2019) or Tarantini et al. (2019). Swing and stance time were not included in these studies. These temporal metrics behave very differently as a function of speed especially at slower walking speeds with swing time changing linearly and stance time logarithmically. Furthermore, we showed that these distinct temporal metrics can be modulated separately by specific CNS circuits [Worley et al., submitted], underlining the importance of analyzing these metrics as this opens avenues for mechanistic, functional-anatomical studies.

Another factor that may further add to differences in results between this and prior studies include the cross-sectional design in prior studies (Tarantini et al., 2019). We followed mice longitudinally after they had reached the mature adult age, and therefore gait metrics in our study are not confounded by differences between cohorts of aged mice, which remained a possibility despite adjustment of metrics for differences in size between animals by Bair et al. Finally, we focused on walking gait, given its translational relevance, while prior studies studied gait that also or mainly included trotting and running based upon the average speeds of 24.6 ± 10 cm/s (Bair et al., 2019) or 50–60 cm/s (Mock et al., 2018).

Velocity dependent relationships of gait metrics have been probed in aging mice (Mock et al., 2018), confirming known speed dependent relationship between gait metrics and speed. However, in contrast to our study, analyses did not correct for speed itself and given that the average gait speed at young age (50–60 cm/s) and old age (30–40 cm/s) was so different, comparisons within the same speed range were not feasible (Mock et al., 2018). We overcame the latter limitations by using a smaller walkway on which mice feel comfortable enough to walk rather than run and by analyzing (similar) gait metrics differently, i.e., by directly comparing gait signatures of the same mice at different ages.

### Strengths and Weaknesses

Strengths of this study include the use of a validated approach which takes into account speed dependence of the gait metrics of interest, an approach that is translatable to human datasets (15). This method was specifically designed to detect whether walking gait metrics that change between conditions are due to a change in speed alone, without a shift in gait signature, or are accompanied by a change in style or gait signature shift. Other strengths are the inclusion of both sexes, and supplementation of gait performance with other measures of mobility. Furthermore, the longitudinal study design allowed us to use the same mice throughout the study, avoiding issues related to sub-strain drift or differences between mice that cannot be captured accurately by models. Additionally, mice were handled by the same tester throughout the study, scoring was performed blinded and we demonstrated reproducibility in two different (male and female) cohorts.

While inclusion of a restricted set of primary gait metrics that dictate speed (stride length, swing, and stance time) is a strength (Lord et al., 2013), there is a multitude of other gait metrics including variability or asymmetry (Broom et al., 2019) that we did not touch upon. Similarly, inclusion of balance and locomotor tests in addition to measures of gait quality provided insights in age-related changes in these modalities during aging, but we did not include other frequently used tests such as rotarod or horizontal ladder. These assays probe motor learning and maintenance of learnt motor skills or motor planning and contrast with the motor tests employed in this study which only required habituation but not training to learn the task. Further studies will be needed to assess the timing and severity of age-related decline in motor learning and planning in relation to the shifts in gait signatures.

Other relative weaknesses include the possibility that the timing of changes in mobility metrics in C57BL6/J mice may not hold in other strains (Wooley et al., 2009). The questions related to sex differences were powered to detect medium to large size effects. Thus, a larger cohort size may reveal additional smaller sex differences. Another relative weakness is that while this study provides insights into aging-related decline in the absence of common neuropathologies, it does not inform on the effects of (combinations of) specific neuropathologies that are common in older adults (see below). Further studies in mouse models that carry key features of neurodegenerative disorders (Lok et al., 2013; Garvock-de Montbrun et al., 2019) will be necessary to reveal the distinctive patterns of gait signature shifts associated with various pathologies and their location as shown for different Parkinson’s models (Broom et al., 2017, 2019).

Finally, an important question remains of whether a decline in balance, locomotor activity or a change in walking style predict mortality or are additional markers for frailty (Baumann et al., 2020). The current study was not powered or designed to answer this question. It ended at 120 weeks, rather than at the humane end point of each mouse. Furthermore, more frequent gait and behavioral assessments would be necessary between the age of maximum weight (79 weeks in our cohort) and the humane end point of each mouse to capture changes in relevant gait metrics that may mark frailty (Kwak et al., 2020).

### Implications for Translation to Older Subjects

This study distinguished between age-related decline in walking speed and a change in the nature of walking as captured by shifts in gait signatures in mice. How are these insights relevant for older adult subjects? Can they be translated to human subjects? The phenomenon of shifts in gait signatures due to CNS perturbations is likely universal across species based on striking similarities in gait curve behavior between experimental parkinsonism in mice and patients with Parkinson’s disease (Broom et al., 2017, 2019). However, an important difference is that in contrast to wild type mouse models (for example C57BL6/J mice), or mouse models that express one or more pathologies, older adult subjects typically develop multiple neuropathologies (Kapasi et al., 2017). Additional non-neurological issues or genetic factors (Ben-Avraham et al., 2017) may further contribute to functional decline in mobility. As a consequence, the direction of changes in gait signatures in the older adult population is unlikely to be as uniform as in mouse models. This adds complexity to human subject research at the group level but opens opportunities for personalized approaches to guide diagnosis and management. For example, distinct patterns of gait signature shifts may point to key pathophysiological factors that include pathology type and region, similar to association between gait speed and regional beta-amyloid (Del Campo et al., 2016). Such insights may aide diagnosis in clinical scenarios and may guide or help monitor effects of individualized intervention.

The dissociation between various mobility measures translates well between mouse and human models. Gait speed (Ko et al., 2010; Buchman et al., 2014; Herssens et al., 2018; Grande et al., 2019), and other components of functional mobility such as balance (Downs et al., 2014) and total daily activity (Buchman et al., 2012) likely represent distinct entities in older adults as they do in mice. These measures should therefore not be used as a proxy for each other (Buchman et al., 2020) in mechanistic studies on aging. However, it is unknown how gait signatures behave in aging human populations. The sudden appearance of shifts in gait signatures, with an increase in stride length and swing time when corrected for speed, was an ominous sign in mice. Further studies in human subjects will be necessary to determine whether the increase in swing time, corrected for gait speed, represents a sensitive predictor of mobility and/or cognitive decline prior to death.

Summarizing, this longitudinal study demonstrated that walking style, quantified by shifts in gait signatures, changed when mice reach the geriatric time point, much later than the decline in walking speed alone or performance in other mobility domains. While the changes in walking style were similar among female and male mice, they developed earlier in females than males. Walking style in geriatric mice contrasts with the styles measured in select parkinsonian neurodegenerative models, illustrating a rich repertoire in walking styles that may signify different underlying mechanisms.

## Data Availability Statement

The raw data supporting the conclusions of this article will be made available by the authors, without undue reservation.

## Ethics Statement

The animal study was reviewed and approved by the Institutional Animal Care and Use Committee at Beth Israel Deaconess Medical Center (experimental protocol #007-2016).

## Author Contributions

LB: design, all behavioral assessments; analyses of balance and open field data sets, and editing of the manuscript. JS and VN: scoring of gait and editing of the manuscript. VV: concept, design, gait analyses, preparation of figures, drafting, and editing of the manuscript. All authors contributed to the article and approved the submitted version.

## Conflict of Interest

The authors declare that the research was conducted in the absence of any commercial or financial relationships that could be construed as a potential conflict of interest.

## Publisher’s Note

All claims expressed in this article are solely those of the authors and do not necessarily represent those of their affiliated organizations, or those of the publisher, the editors and the reviewers. Any product that may be evaluated in this article, or claim that may be made by its manufacturer, is not guaranteed or endorsed by the publisher.
