# Supplementary material for: Shifts in Gait Signatures Mark the End of Lifespan in Mice, With Sex Differences in Timing
Source: Front Aging Neurosci. 2021 Aug 2;13:716993. doi: 10.3389/fnagi.2021.716993 (PMC8366415; doi:10.3389/fnagi.2021.716993)
Supplement: Supplementary file 1 [file Data_Sheet_1.pdf]

Table S1 Age-related changes in gait metrics

Accompanying Fig. 2

Gait signatures

N=15	Extra sum of squares F test F (DFn, DFd)	P Value alpha 0.001
**Week 19 vs Week 55**		
stride length	0.9547 (2, 897)	0.39
swing time	0.5463 (2, 897)	0.58
log stance time	1.813 (2, 1056)	0.16
cadence	1.345 (2, 811)	0.26
**Week 19 vs Week 99**		
stride length	1.479 (2, 866)	0.23
swing time	1.631 (2, 866)	0.20
log stance time	3.559 (2, 1008)	0.03
cadence	4.893 (2, 778)	0.008
**Week 19 vs Week 120**		
stride length	18.58 (2, 868)	<0.0001
swing time	26.51 (2, 868)	<0.0001
log stance time	15.15 (2, 1005)	<0.0001
cadence	36.42 (2, 798)	<0.0001

Averaged metrics

RM-one-way ANOVA, linear mixed effects model N=15	F	P Value alpha 0.05	Dunnett's multiple comparisons test Week	Mean Diff.	95.00% CI of diff.	Adjusted P Value
speed	F (2.047, 38.21) = 9.047	0.0009	19vs. 55	2.583	0.5969 to 4.569	0.01
		0.0006	19 vs. 99	2.489	0.3767 to 4.601	0.02
			19 vs. 120	3.564	1.052 to 6.075	0.006
weight	F (1.704, 23.85) = 28.61	<0.0001	19vs. 55	-8.533	-12.00 to -5.071	<0.0001
		<0.0001	19 vs. 99	-11.67	-16.46 to -6.873	<0.0001
			19 vs. 120	-7.8	-12.19 to -3.411	0.001
stride length	F (2.698, 37.77) = 1.916	0.15				
swing time	F (2.462, 34.47) = 7.650	0.0009	19vs. 55	0.008696	-0.004348 to 0.02174	0.23
		0.0009	19 vs. 99	0.00765	-0.005177 to 0.02048	0.31
			19 vs. 120	-0.01079	-0.02335 to 0.001770	0.10
stance time	F (1.842, 34.39) = 3.959	0.04	19vs. 55	-0.08116	-0.1626 to 0.0002466	0.051
		0.03	19 vs. 99	-0.04661	-0.1484 to 0.05515	0.50
			19 vs. 120	-0.1434	-0.2894 to 0.002600	0.055
cadence	F (2.102, 39.24) = 11.29	0.0004	19vs. 55	0.4012	0.05756 to 0.7448	0.02
		0.0001	19 vs. 99	0.3454	-0.06447 to 0.7552	0.11
			19 vs. 120	0.7187	0.2812 to 1.156	0.002

Table S2 Age-related changes in gait metrics in female mice

Accompanying Fig. 3

Gait signatures	Cohort up to week 120	
FEMALE N=7	Sum of squares F test<br>F (DFn, DFd)	P Value<br>alpha 0.001
**Week 19 vs Week 55**		
stride length	2.046 (2, 430)	0.13
swing time	1.280 (2, 430)	0.28
log stance time	0.3713 (2, 523)	0.69
cadence	1.616 (2, 387)	0.20
**Week 19 vs Week 99**		
stride length	2.727 (2, 385)	0.07
swing time	0.2842 (2, 385)	0.75
log stance time	7.912 (2, 467)	0.0004
cadence	5.878 (2, 341)	0.003
**Week 19 vs Week 120**		
stride length	8.010 (2, 390)	0.0004
swing time	10.40 (2, 390)	<0.0001
log stance time	8.087 (2, 469)	0.0004
cadence	17.17 (2, 341)	<0.0001

Averaged metrics	Cohort up to week 120		Dunnett's multiple comparisons test			
FEMALE N=7	RM-one-way ANOVA<br>F	P Value<br>alpha 0.05	Week	Mean Diff.	95.00% CI of diff.	Adjusted<br>P Value
weight	F (2.025, 12.15) = 47.62	<0.0001	19 versus 55	-11.57	-15.84 to -7.302	0.0004
			19 versus 99	-16.29	-21.85 to -10.72	0.0003
			19 versus 120	-12.71	-18.60 to -6.829	0.001
speed	F (1.505, 9.031) = 4.68	0.048	19 versus 55	3.733	-0.1495 to 7.616	0.06
			19 versus 99	3.657	-0.4734 to 7.787	0.08
			19 versus 120	3.355	-2.142 to 8.851	0.23
stride	F (1.849, 11.09) = 1.68	0.23				
swing	F (2.435, 14.61) = 1.649	0.22				
stance	F (2.275, 13.65) = 2.392	0.12				
cadence	F (1.617, 9.703) = 4.014	0.06				

Gait signatures	Cohort up to week 99	
FEMALE N=14	Sum of squares F test<br>F (DFn, DFd)	P Value<br>alpha 0.001
**Week 19 vs Week 55**		
stride length	2.968 (2, 842)	0.05
swing time	4.545 (2, 842)	0.01
log stance time	2.257 (2, 1086)	0.11
cadence	2.794 (2, 756)	0.06
**Week 19 vs Week 99**		
stride length	6.222 (2, 735)	0.002
swing time	0.3134 (2, 735)	0.73
log stance time	11.96 (2, 925)	<0.0001
cadence	11.16 (2, 637)	<0.0001

Averaged metrics	Cohort up to week 99		Dunnett's multiple comparisons test			
	RM-one-way ANOVA<br>F	P Value<br>alpha 0.05	Weeks	Mean Diff.	95.00% CI of diff.	Adjusted<br>P Value
speed	F (1.389, 18.06) = 31.51	<0.0001	19 versus 55	4.492	2.572 to 6.413	0.0001
			19 versus 99	5.102	3.02 to 7.184	0.0001

Table S3 Age-related changes in gait metrics in male mice

Accompanying Fig. 4

Gait signatures<br>MALE N=8	Cohort up to week 120<br>Sum of squares F test<br>F (DFn, DFd)	P Value<br>alpha 0.001
**Week 19 vs Week 55**		
stride length	2.036 (2, 463)	0.13
swing time	3.899 (2, 463)	0.02
log stance time	2.390 (2, 529)	0.09
cadence	1.441 (2, 420)	0.24
**Week 19 vs Week 99**		
stride length	0.008818 (2, 477)	0.99
swing time	1.800 (2, 477)	0.17
log stance time	2.010 (2, 537)	0.14
cadence	0.4483 (2, 433)	0.64
**Week 19 vs Week 120**		
stride length	10.36 (2, 474)	<0.0001
swing time	16.47 (2, 474)	<0.0001
log stance time	7.357 (2, 532)	0.0007
cadence	19.34 (2, 453)	<0.0001

Averaged metrics<br>MALE N=8	Cohort up to week 120<br>RM-one-way ANOVA<br>F	P Value<br>alpha 0.05	Dunnett's multiple comparisons test<br>Week	Mean Diff.	95.00% CI of diff.	Adjusted<br>P Value
weight	F (3, 21) = 8.698	0.0006	19 versus 55	-5.875	-9.879 to -1.871	0.004
			19 versus 99	-7.625	-11.63 to -3.621	0.0003
			19 versus 120	-3.5	-7.504 to 0.5040	0.09
speed	F (3, 21) = 7.326	0.002	19 versus 55	1.577	-0.4645 to 3.618	0.15
			19 versus 99	1.467	-0.5743 to 3.508	0.20
			19 versus 120	3.746	1.705 to 5.788	0.0004
stride length	F (3, 21) = 1.431	0.26				
swing time	F (3, 21) = 8.548	0.0007	19 versus 55	0.01452	0.001056 to 0.02798	0.03
			19 versus 99	0.0105	-0.002965 to 0.02396	0.15
			19 versus 120	-0.009935	-0.02340 to 0.003528	0.18
stance time	F (3, 21) = 5.370	0.007	19 versus 55	-0.04541	-0.1979 to 0.1071	0.79
			19 versus 99	0.01119	-0.1413 to 0.1637	0.995
			19 versus 120	-0.2028	-0.3553 to -0.05023	0.008
cadence	F (3, 21) = 11.33	0.0001	19 versus 55	0.2871	-0.07498 to 0.6491	0.14
			19 versus 99	0.1197	-0.2424 to 0.4817	0.74
			19 versus 120	0.7745	0.4124 to 1.137	<0.0001

Gait signatures<br>MALE N=11	Cohort up to week 99<br>Sum of squares F test<br>F (DFn, DFd)	P Value<br>alpha 0.001
**Week 19 vs Week 55**		
stride length	3.72 (2, 627)	0.02
swing time	2.133 (2, 627)	0.12
log stance time	6.865 (2, 739)	0.001
cadence	4.347 (2, 559)	0.01
**Week 19 vs Week 99**		
stride length	0.9876 (2, 663)	0.37
swing time	1.27 (2, 663)	0.28
log stance time	1.929 (2, 766)	0.15
cadence	1.493 (2, 602)	0.23

Averaged metrics	Cohort up to week 99<br>RM-one-way ANOVA<br>F	P Value<br>alpha 0.05	Dunnett's multiple comparisons test<br>Weeks	Mean Diff.	95.00% CI of diff.	Adjusted<br>P Value
speed	F (1.655, 16.55) = 6.203	0.013	19 versus 55	1.644	0.03491 to 3.253	0.05
			19 versus 99	1.905	0.1837 to 3.627	0.03

Table S4 Sex differences in gait metrics at key time points during aging

Accompanying Fig. 5

Gait signatures		Male versus female<br>Sum of squares F test<br>F (DFn, DFd)	P Value<br>alpha 0.001
**Stride length**			
	week 19	2.363 (2, 652)	0.09
	week 55	3.694 (2, 815)	0.03
	week 99	15.60 (2, 744)	<0.0001
	week 120	0.2489 (2, 467)	0.78
**Swing time**			
	week 19	4.137 (2, 641)	0.02
	week 55	33.47 (2, 815)	<0.0001
	week 99	12.37 (2, 742)	<0.0001
	week 120	5.605 (2, 467)	0.004
**Stance time**			
	week 19	3.481 (2, 891)	0.03
	week 55	11.43 (2, 927)	<0.0001
	week 99	10.85 (2, 793)	<0.0001
	week 120	3.009 (2, 494)	0.05
**Cadence time**			
	week 19	0.2748 (2, 530)	0.76
	week 55	3.879 (2, 781)	0.02
	week 99	9.705 (2, 705)	<0.0001
	week 120	0.1516 (2, 456)	0.86

Table S5 **Summary of mobility measures in male and female mice**

Accompanying Fig. 6

	RM-one-way ANOVA F	P Value alpha 0.05	Dunnett's multiple comparisons test Weeks	Mean Diff.	95.00% CI of diff.	Adjusted P Value
Weight	F (1.263, 16.42) = 70.83	<0.0001	19 versus 55	-11.57	-14.05 to -9.097	0.0001
			19 versus 99	-18.29	-23.33 to -13.24	0.0001
Beam	F (1.603, 9.615) = 16.79	0.001	19 vs. 29	0.7143	-5.362 to 6.791	0.97
			19 vs. 55	-3.714	-9.902 to 2.474	0.24
			19 vs. 94	-16.14	-27.39 to -4.898	0.01
Immobile (Open field)	F (1.837, 23.89) = 9.456	0.0012	19 versus 55	-101.7	-197.2 to -6.23	0.04
			19 versus 99	-199.9	-322.1 to -77.69	0.003
Distance (Open Field)	F (1.941, 25.24) = 5.685	0.0097	19 versus 55	7.021	-2.912 to 16.95	0.18
			19 versus 99	12.45	3.568 to 21.34	0.008

	RM-one-way ANOVA F	P Value alpha 0.05	Dunnett's multiple comparisons test Weeks	Mean Diff.	95.00% CI of diff.	Adjusted P Value
Weight	F (1.515, 15.15) = 14.31	0.0007	19 versus 55	-5.636	-8.909 to -2.364	0.002
			19 versus 99	-7	-11.44 to -2.565	0.004
Beam	F (3, 18) = 16.81	<0.0001	19 vs. 29	-3.429	-12.82 to 5.968	0.68
			19 vs. 55	-10.71	-20.11 to -1.318	0.02
			19 vs. 94	-24	-33.40 to -14.60	<0.0001
Immobile (Open field)	F (1.282, 12.82) = 12.1	0.0027	19 versus 55	33.34	-34.46 to 101.1	0.38
			19 versus 99	-173.6	-322.1 to -25.15	0.02
Distance (Open Field)	F (1.143, 11.43) = 5.184	0.039	19 versus 55	2.751	-0.9345 to 6.437	0.15
			19 versus 99	8.461	1.814 to 15.11	0.02

Table S6 **Effect of sex on mobility measures during aging**

Accompanying text

ANOVA table		2-Way-ANOVA F (DFn, DFd)	P Value alpha 0.05	Sidak's multiple comparisons test Male vs Female at weeks:	Mean Diff.	95.00% CI of diff.	Adjusted P Value
**Weight**							
	Sex x Age	F (2, 46) = 13.86	<0.0001	19	7.545	5.736 to 9.355	<0.0001
	Sex	F (1, 23) = 1.758	0.20	55	1.61	-2.143 to 5.364	0.63
	Age	F (1.373, 31.58) = 72.55	<0.0001	99	-3.74	-10.54 to 3.060	0.43
**Immobile (Open field)**							
	Sex x Age	F (2, 46) = 0.8077	0.45	19	14.35	-42.68 to 71.37	0.89
	Sex	F (1, 23) = 0.07636	0.78	55	-54.01	-157.7 to 49.73	0.47
	Age	F (1.664, 38.26) = 19.98	<0.0001	99	21.44	-128.5 to 171.4	0.98
**Beam**							
	Sex x Age	F (2, 36) = 3.426	0.04	19	2.136	-5.813 to 10.09	0.87
	Sex	F (1, 23) = 5.513	0.03	55	15.21	1.257 to 29.17	0.03
	Age	F (1.431, 25.76) = 16.03	0.0001	99	4.519	-11.88 to 20.92	0.85
**Stride Velocity**							
	Sex x Age	F (3, 52) = 1.994	0.13	19	-1.734	-6.265 to 2.797	0.72
	Sex	F (1, 52) = 2.334	0.13	55	0.4228	-1.269 to 2.115	0.91
	Age	F (1.813, 31.43) = 9.811	0.0007	99	0.4561	-1.472 to 2.385	0.94
				120	-2.126	-5.008 to 0.7564	0.20

Table S7 Correlation between changes in performance in gait and mobility

Accompanying text

Pearson r correlation matrix

N=15 (N=7 females and N=8 males)

**Pearson r**	Beam wk 55	Open field wk 99	Speed wk 120	Swing time wk 120
Beam wk 55	1	0.13	-0.15	0.07
Open field wk 99	0.13	1	-0.08	0.42
Speed wk 120	-0.15	-0.08	1	-0.22
Swing time wk 120	0.07	0.42	-0.22	1
**p value (alpha 0.05)**				
Beam wk 55		0.65	0.59	0.80
Open field wk 99	0.65		0.78	0.12
Speed wk 120	0.59	0.78		0.44
Swing time wk 120	0.80	0.12	0.44	
